# The effects of climatic and non-climatic factors on malaria mortality at different spatial scales in western Kenya, 2008–2019

**DOI:** 10.1136/bmjgh-2023-014614

**Published:** 2024-09-07

**Authors:** Bryan O. Nyawanda, Sammy Khagayi, David Obor, Steve B. Odhiambo, Anton Beloconi, Nancy A. Otieno, Godfrey Bigogo, Simon Kariuki, Stephen Munga, Penelope Vounatsou

**Affiliations:** 1 Centre for Global Health Research, Kenya Medical Research Institute, Kisumu, Kenya; 2 Swiss Tropical and Public Health Institute, Allschwil, Switzerland; 3 University of Basel, Basel, Switzerland

**Keywords:** Malaria, Descriptive study, Kenya, Medical demography, Epidemiology

## Abstract

**Background:**

Malaria mortality is influenced by several factors including climatic and environmental factors, interventions, socioeconomic status (SES) and access to health systems. Here, we investigated the joint effects of climatic and non-climatic factors on under-five malaria mortality at different spatial scales using data from a Health and Demographic Surveillance System (HDSS) in western Kenya.

**Methods:**

We fitted Bayesian spatiotemporal (zero-inflated) negative binomial models to monthly mortality data aggregated at the village scale and over the catchment areas of the health facilities within the HDSS, between 2008 and 2019. First order autoregressive temporal and conditional autoregressive spatial processes were included as random effects to account for temporal and spatial variation. Remotely sensed climatic and environmental variables, bed net use, SES, travel time to health facilities, proximity from water bodies/streams and altitude were included in the models to assess their association with malaria mortality.

**Results:**

Increase in rainfall (mortality rate ratio (MRR)=1.12, 95% Bayesian credible interval (BCI): 1.04–1.20), Normalized Difference Vegetation Index (MRR=1.16, 95% BCI: 1.06–1.28), crop cover (MRR=1.17, 95% BCI: 1.11–1.24) and travel time to the hospital (MRR=1.09, 95% BCI: 1.04–1.13) were associated with increased mortality, whereas increase in bed net use (MRR=0.84, 95% BCI: 0.70–1.00), distance to the nearest streams (MRR=0.89, 95% BCI: 0.83–0.96), SES (MRR=0.95, 95% BCI: 0.91–1.00) and altitude (MRR=0.86, 95% BCI: 0.81–0.90) were associated with lower mortality. The effects of travel time and SES were no longer significant when data was aggregated at the health facility catchment level.

**Conclusion:**

Despite the relatively small size of the HDSS, there was spatial variation in malaria mortality that peaked every May–June. The rapid decline in malaria mortality was associated with bed nets, and finer spatial scale analysis identified additional important variables. Time and spatially targeted control interventions may be helpful, and fine spatial scales should be considered when data are available.

**WHAT IS ALREADY KNOWN ON THIS TOPIC**

- Malaria mortality has been declining over time, a phenomenon largely attributed to control interventions but threatened by climate change.
- Varied results have been reported when investigating association between malaria mortality and climatic or non-climatic factors.

**WHAT THIS STUDY ADDS**

- The joint effects of climatic, interventions and other non-climatic factors are not fully explored and may differ from one setting to another.
- Additionally, there are very few studies that have assessed the impact of data aggregation on observed results.
- This study explored the joint effect of several covariates and elucidated the impact of data aggregation at different spatial scales.

**HOW THIS STUDY MIGHT AFFECT RESEARCH, PRACTICE OR POLICY**

- This study highlights areas of high malaria mortality.
- These areas may require targeted control interventions.
- Furthermore, this study highlights the influence of spatial scales on covariate effects.
- This finding may be useful to researchers conducting spatial analyses; they should consider nuanced spatial scales and Bayesian inference, which incorporates spatial variation and correlation.

## Background

 Despite notable strides made in reducing malaria morbidity and mortality since the year 2000, the disease still remains one of the leading causes of mortality, especially among children aged<5 years. The WHO estimated that globally malaria caused 608 000 deaths in 2022 and 96% (approximately 583 680) of the deaths occurred in the African region.^1^ Similar to the global trend, Kenya has observed a decline in malaria burden over time.^2^,^5^ This has majorly been attributed to the upscaling of malaria control programmes, including the distribution of insecticide-treated bed nets (ITNs), intermittent preventive treatment in pregnancy (IPTp) and prompt diagnosis and management of malaria cases.^3^ This reduction may also be attributed to the implementation of integrated management of childhood illness (IMCI) in 1996/1997 and integrated community case management (iCCM) in 2013.^6^,^8^ IMCI involves strengthening health worker skills, strengthening health systems and improving community health practices, whereas iCCM involves providing community health workers (CHWs) with a basic health package, which includes malaria rapid diagnostic testing kits and drugs for community-level management of malaria. To further reduce malaria morbidity and mortality, Kenya developed the *Kenya Malaria Strategy (KMS) 2019–2023* which targeted to reduce both malaria incidence and deaths by 75% of 2016 levels by 2023.^3^

The female *Anopheles* mosquito is the main vector for malaria transmission. The abundance of this vector depends on environmental suitability—which is mainly driven by climate, and vector control measures in place. The main climatic drivers of malaria transmission are rainfall, humidity and temperature.^9^ Other environmental factors associated with malaria include altitude, land cover and use, and distance from potential breeding sites. Though climatic and environmental factors are often summarised at various scales, their distribution varies even in small geographical areas. Previous studies in other fields have shown that there are differences in estimates under different spatial aggregation scales.^10^,^12^ However, little has been done to elucidate the potential effect of spatial aggregation scales on malaria mortality model estimates, partly due to lack of good quality spatiotemporal data. Data from Health and Demographic Surveillance Systems (HDSS) provides an opportunity to investigate the relative effect of climatic and non-climatic factors on malaria mortality at different spatial scales.

The Kenya Medical Research Institute (KEMRI) and the US Centers for Disease Control and Prevention (CDC) run a HDSS in western Kenya. Previous studies in this HDSS observed that malaria was the leading cause of death among children aged<5 years.^5 13^ Earlier studies conducted in this HDSS associated bed net use with a reduction in both morbidity and mortality,^14 15^ while Ombok *et al* estimated mortality over space using classical statistical methods and observed associations between malaria deaths and some geospatial factors including distance to streams and population densities.^16^ More recently, malaria mortality was characterised by age,^4^ linked to malaria transmission,^5 17 18^ climate variability^19^ and other non-climatic factors within this HDSS.^20^ That notwithstanding, these studies had their limitations. Either they analysed mortality counts without considering the duration of exposure, or they did not adjust for climatic/environmental factors or other non-climatic drivers of malaria mortality, such as interventions—bed net use and iCCM, socioeconomic status (SES), and access and utilisation of health systems. Furthermore, apart from a few studies,^5 17 18^ most of the previous work relied on classical statistical methods, therefore, potential variations and correlations of malaria mortality existing in space and time were not taken into consideration. Consequently, the estimates of the effects of spatiotemporal covariates were potentially biased.

Elsewhere, studies have independently assessed the effects of interventions or climate variability on malaria mortality,^21 22^ including one in the Democratic Republic of Congo that forecasted malaria morbidity and mortality.^23^ There is need for inclusion of both climatic and non-climatic variables while modelling future malaria severity and mortality scenarios to inform health systems preparedness. Higher health facility readiness has been shown to reduce the risk of severe malaria outcomes.^24^ Additionally, understanding the effect of spatial scales on estimates may be beneficial to researchers. Bayesian model formulations allow for inclusion of various covariates while accounting for potential variation and correlation in space and time.^25^

In this study, we used Bayesian spatiotemporal models to estimate the effects of climate variability, interventions and other non-climatic factors on the spatiotemporal patterns of under-five malaria mortality in the western Kenya HDSS. The analysis was carried out at two geographical scales, that is, at the individual village level and the health facility catchment level from 2008 to 2019.

## Methods

### Study area

**Figure 1:**
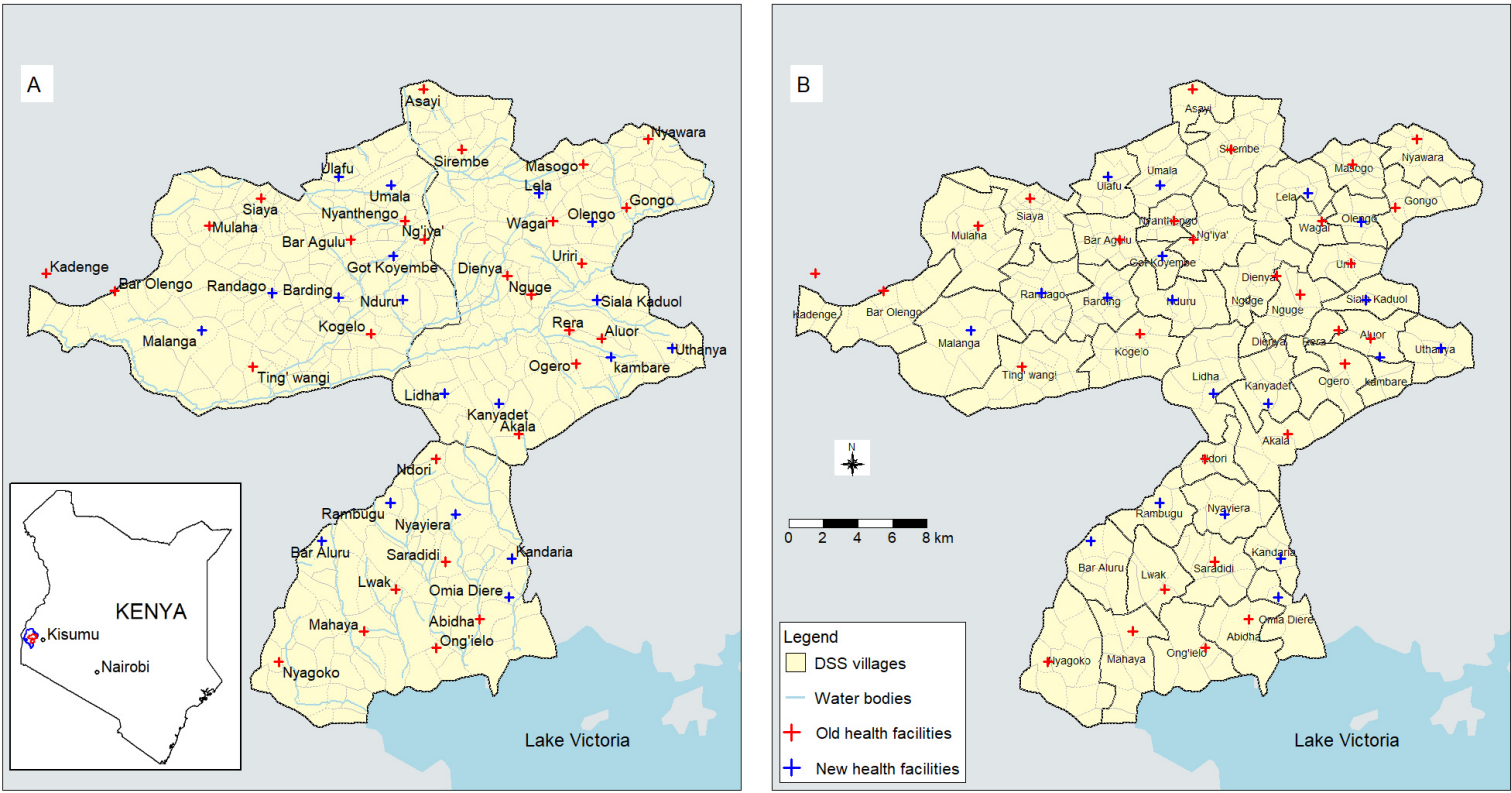
Map of the western Kenya Health and Demographic Surveillance System (HDSS) villages and sentinel health facilities. The three HDSS areas Asembo, Gem and Karemo are displayed in the lower, upper right and upper left dark boundaries, respectively (A). The hospital catchment areas (B) and the inset shows the location of Siaya county (blue) and the HDSS (red).

The KEMRI-CDC HDSS spans three areas; Asembo, Gem and Karemo, located in Rarieda, Gem and Alego Usonga subcounties, respectively (figure 1). The HDSS covers approximately 700 km^2^ in Siaya county, western Kenya. The burden of malaria and HIV is high in this area compared with other parts of Kenya.^26 27^ The KEMRI-CDC HDSS follows a population of over 240 000 individuals and documents demographic and socioeconomic data, including births, deaths, migrations, education levels, bed net ownership and use, and variables for the estimation of SES. KEMRI in partnership with the CDC also conducts population-based infectious disease surveillance (PBIDS) for infectious diseases within 33 HDSS villages in Asembo. We used data from these two surveillance platforms. More detailed information about this HDSS and PBIDS have been previously described.^28^,^30^

### Sources of data

#### Malaria mortality data

Causes of death (COD) in the HDSS are determined through verbal autopsies (VA) on all deaths as previously described.^29^ We used VA data collected from children aged<5 years during 2008–2019 in this analysis. COD, including malaria deaths, were inferred using the InterVA-4 method. This probabilistic method uses Bayesian approaches to assign the three most probable COD using medical history and signs and symptoms exhibited by the deceased before death.^31^ No malaria-associated neonatal deaths were reported during the study period.

#### Climatic/environmental data

We extracted daytime land surface temperature (LSTD) at a 1×1 km^2^ spatial and 8-day temporal resolution and Normalized Difference Vegetation Index (NDVI) at a 1×1 km^2^ spatial and 16-day temporal resolution from the Moderate Resolution Imaging Spectroradiometer on board NASA’s Terra and Aqua satellites.^32 33^ Rainfall estimates were extracted from the Climate Hazards Group InfraRED Precipitation with Station data at 5.6×5.6 km^2^ spatial and 5-day temporal resolutions.^34^ These data were processed in Google Earth engine at 1×1 km^2^ spatial and monthly temporal resolution. Night-time land surface temperature was not used in this analysis because we previously found that it was not associated with malaria incidence in this setting.^35^ The average altitude for each village was estimated using the digital elevation model obtained from the Shuttle Radar Topographic Mission at 90 m spatial resolution,^36^ while land cover data was obtained from Copernicus at 100×100 m^2^^37^ and surface water coverage obtained from the Joint Research Centre at 30×30 m^2^.^38^ The datasets were downloaded as raster images from Google Earth engine, then clipped and extracted using the villages shapefiles in R software (Vienna, Austria).

#### Other data

Other data used in this analysis includes distance to the nearest health facility, SES, bed net use and years when iCCM was implemented. Distances between household locations and health facilities were calculated as the travel distance using gmapdistance package^39^ in R, which uses Google Maps, assuming that the participants walk to the hospital (motorcycles and bicycles, which are commonly used for transport in the area, use similar paths). This analysis included 45 public health facilities in the HDSS and three (Lwak, Aluor and Ngi’ya) faith-based facilities that are part of the HDSS surveillance (figure 1). Distances to the nearest health facility were redefined for each household per when new health facilities were established. Most of these new health facilities were established between 2010 and 2011. Euclidean distances from household locations to the nearest stream were also calculated. Annual SES indices for each village were generated from the socioeconomic indicators data that the HDSS collects every 2 years from enrolled households using multiple correspondence analysis as described previously.^40^ Bed net use data from all 384 villages were available until 2013. The HDSS stopped collecting this information thereafter, except in 33 villages in the area where PBIDS surveillance occurs (subset of Asembo area). We observed that the distribution of bed nets use was similar in the three areas (Asembo, Gem and Karemo) during 2011–2013 after the 2011 mass ITN distribution. For this reason, we aggregated bed net use data from the 33 villages during 2014–2019 and assigned it to all the villages after 2013. While IMCI was adopted in 1996/1997^8^ and artemisinin-based combination therapy (ACT) was scaled up in 2006^41^ before the start of our study, iCCM was implemented in 2013.^7^ We therefore did not include IMCI and ACT use in the analysis, as this was the standard of care covering the entire study period. However, iCCM was included from 2013. Indoor residual spraying (IRS) and seasonal chemoprevention were not implemented in this area, and therefore, not considered.

### Statistical analysis

The number of malaria deaths in a given village and month was divided by the total monthly person-years of observation (pyo) of the village to calculate village-specific and month-specific under-five malaria mortality rates. Person time was defined as the time spent in the HDSS from enrolment (some through birth) to exit via death, out-migration, loss to follow-up or end of the study period (31 December 2019 for this analysis).

There is a time delay (lag time) between climatic suitability and malaria transmission. In our previous work,^35^ using Bayesian variable selection, we showed that the variability in malaria incidence in this area was best explained by rainfall and temperature lagged over the previous 2 and 1 month, respectively. In this article, we used a goodness of fit measure, namely the deviance information criterion (DIC), to determine the model with the best rainfall, temperature and NDVI lag combination associated with malaria mortality. In particular, we examined the current month of mortality (lag0), the previous month (lag1) and the month before that (lag2). The model with the smallest DIC was regarded to have the best lag combination.

Before fitting Bayesian models, Pearson correlation was used to assess the relationship between lagged climatic variables with mortality. Collinearity between the predictors was assessed based on variance inflation factor (VIF). Bayesian zero-inflated negative binomial (ZINB) and negative binomial (NB) models with first order autoregressive (AR^1^) temporal and conditional autoregressive spatial processes were then fitted to determine the association between the selected climatic and non-climatic variables with malaria mortality at the village level. ZINB model was chosen for the village-level analysis due to the excess of zero values (95%) for some months and villages, whereas NB models were fitted at the village level for validation purposes. Temporal and spatial random effects were included in the models to account for the spatiotemporal correlation in malaria deaths. All the continuous covariates were standardised to allow direct comparison of their coefficients. The detailed model formulation is described in online supplemental material 1.

An additional analysis was carried out to evaluate the impact of geographical scale in the estimation of covariate effects on malaria mortality. More specifically, we aggregated the mortality data over health facility catchment areas and fitted a NB—instead of the ZINB model. The climatic and non-climatic factors were averaged within the health facility catchment areas. To assess the impact of the categorical form of temperature, separate models were fitted considering cut-offs of <25°C, 25–30°C and >30°C. The best model (lag combination) was determined by comparing the DIC and the root mean square error (RMSE).

Time-series plots were used to describe the monthly (seasonal) and annual variation of malaria mortality, climatic and non-climatic factors in the HDSS during the study period, whereas spatial maps were used to display their variation in space. Smoothing splines with 10 knots were used to fit the annual trends. Pie charts were used to display the distribution of COD, and R V.4.0.2^42^ was employed for data management and statistical analysis. The model parameters were estimated using Markov chain Monte Carlo simulations in the Just Another Gibbs Sampler software.

## Results

### Descriptive statistics

Malaria was responsible for approximately one in every three deaths (36.76%) among children aged<5 years in the 384 villages in the KEMRI-CDC HDSS from 2008 to 2019 (online supplemental figure S1). Malaria deaths decreased by 26% between 2008 (40.95%) and 2014 (30.17%) and slightly increased in 2019 (31.46%) compared with 2014.

**Figure 2:**
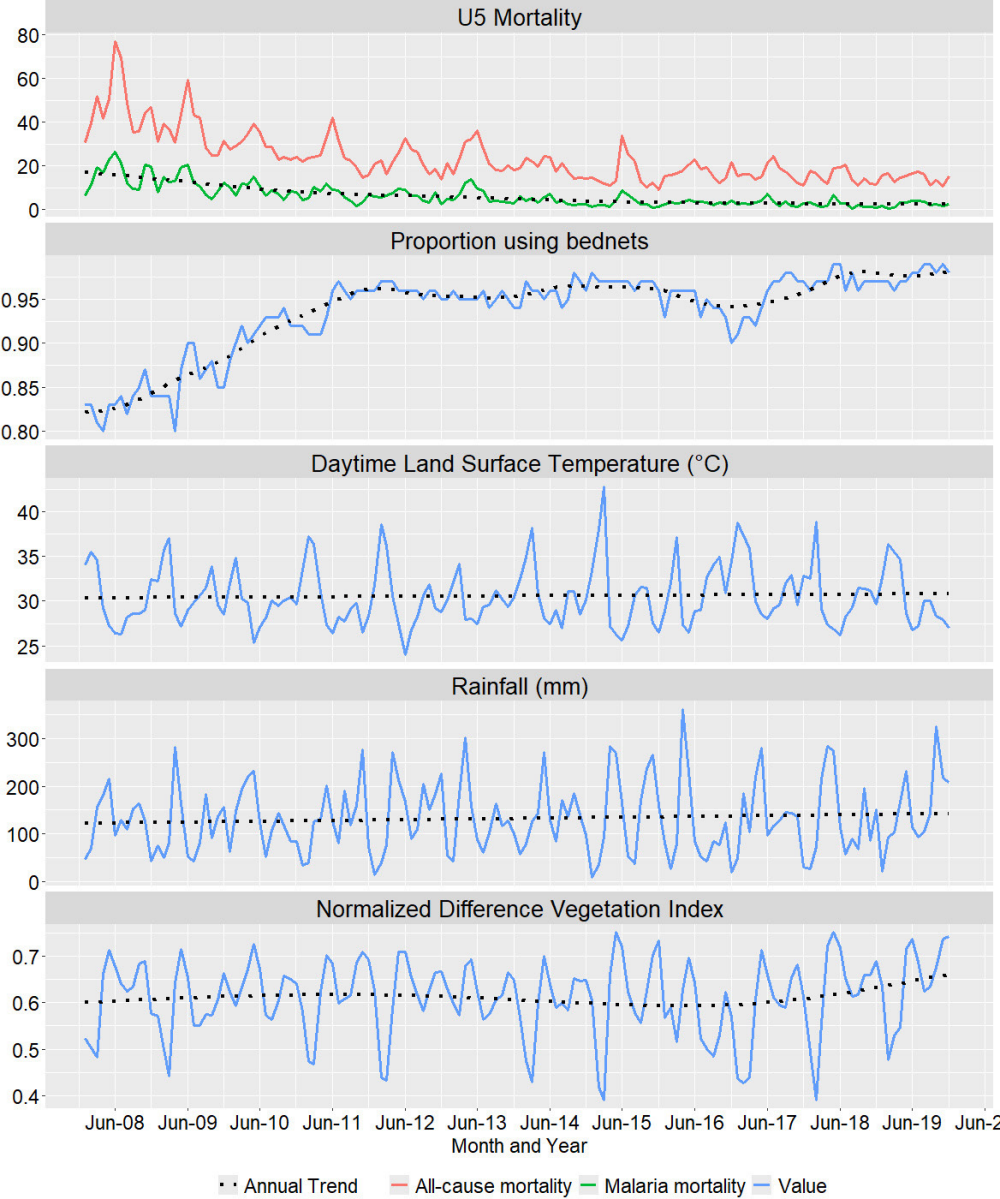
Monthly time-series plots and annual trends of all-cause and malaria-specific mortality per 1000 person-years, the proportion of individuals who slept under bed nets in the population-based infectious disease surveillance area, the mean daytime land surface temperature, the mean rainfall and the mean Normalized Difference Vegetation Index from 2008 to 2019.

**Figure 3:**
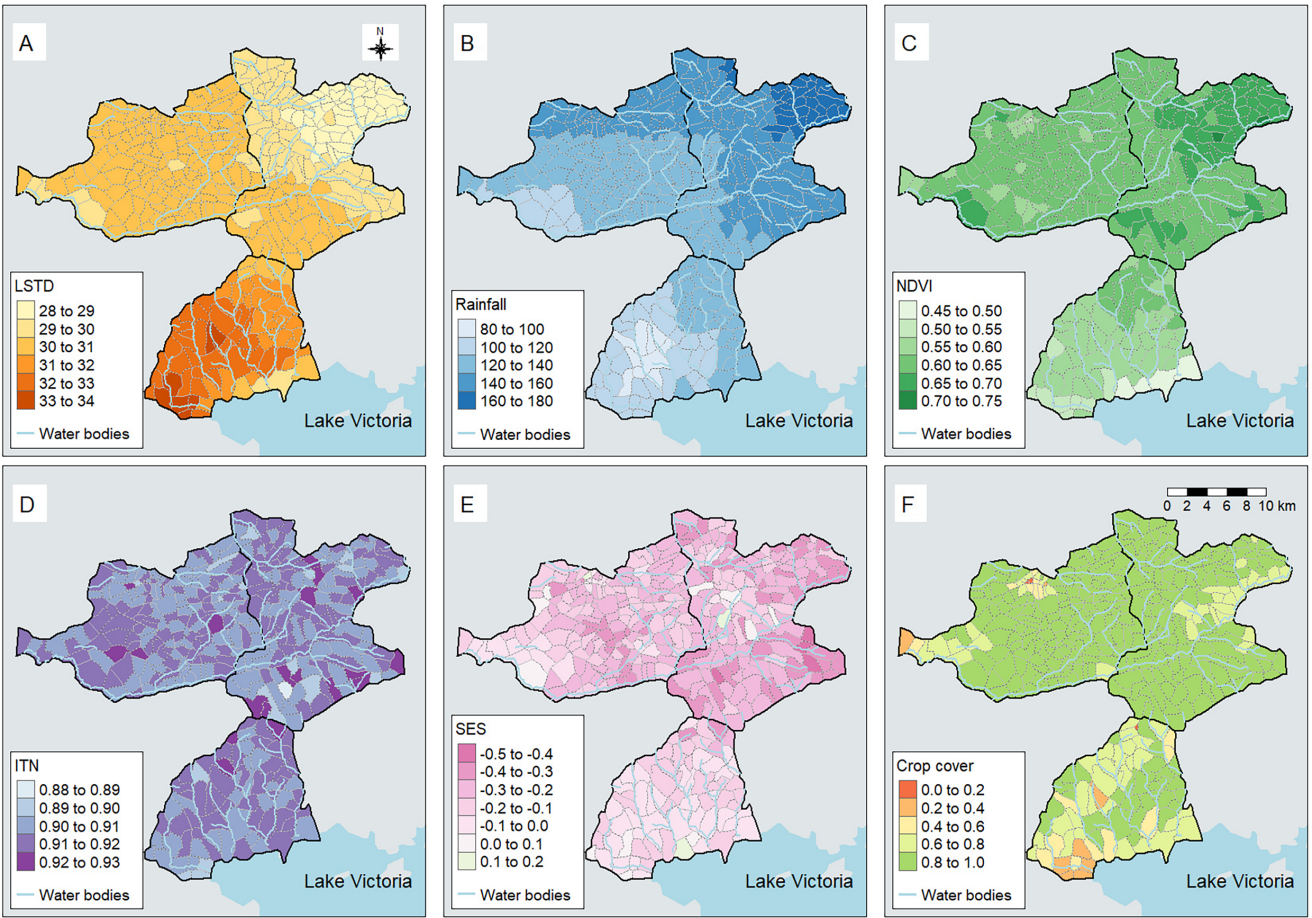
Spatial distribution of averaged daytime land surface temperature (LSTD) (A), rainfall (B), Normalized Difference Vegetation Index (NDVI) (C), bed net use (D), socioeconomic status (SES) (E) and crop cover (F) over the Health and Demographic Surveillance System from 2008 to 2019. Bed net use averaged from 2011 to 2013.

Figure 2 displays the monthly time-series of all-cause and malaria-specific under-five mortality per 1000 pyo, of the proportion of under-fives sleeping under bed net, of average LSTD and of average rainfall in the HDSS. A total of 3056 malaria deaths were reported among children aged<5 years over 479 213 pyo between 2008 and 2019. Every year, May and June had the highest mortality rates, while October had the lowest mortality rates. The mortality peak mostly occurred 1 month before the incidence peak (online supplemental figure S2). The highest (36.0°C) and lowest (27.0°C) average monthly LSTD were observed in February and June, whereas the highest and lowest average monthly rainfall were observed in April (237 mm) and January (44 mm), respectively. NDVI peaks occurred 1 month after rainfall peaks. There was a noticeable increase in bed net coverage and use from 2008 (83%) to 2019 (97%). Asembo was warmer, had lesser rainfall, lower vegetation index and lesser crop cover compared with Gem and Karemo (figure 3). Bed net use was uniform across the three areas, while SES was lower in Gem.

The annual trends in malaria mortality, LSTD, rainfall, NDVI and bed net use are shown in figure 2. The highest annual malaria mortality rate among children aged<5 years was observed in 2008 (16.35 per 1000 pyo) and the lowest in 2018 (2.14 per 1000 pyo). Malaria mortality decreased significantly between 2008 and 2011, but the decline was slow thereafter. All-cause mortality followed a similar trend as malaria mortality. The median altitude of the villages in the DSA was 1307 m (1149–1454 m) and the median walking travel time to the nearest health facility was 0.89 (0.13–2.90) hours during 2008–2010, and 0.60 (0.10–1.81) hours thereafter. The median distance to the nearest water body was 0.75 (0.14–3.14) km.

### Selection of climatic variable lags

Correlation coefficients between lagged climatic factors and malaria mortality at health facility catchment and village levels are presented in online supplemental table S1. Generally, the strongest relationship between LSTD and NDVI occurred in the same month, whereas the strongest relationship between both LSTD and NDVI with rainfall was observed with increase in rainfall in the preceding 1 month. VIF was smaller than 5, therefore there was no collinearity, hence no variable was dropped. At the health facility catchment level, rainfall, LSTD and NDVI lagged over the previous 1 month had the lowest DIC of 10 349 (table 1). Similar model fits were observed for different lag combinations when LSTD was categorised (online supplemental table S2), therefore, the continuous form was considered. For the village-level analysis, the same combination returned a lower DIC (37592). For the validation of the ZINB model, we observed that it had slightly lower RMSE compared with NB models (2.58 vs 2.78) for rainfall lag1 and LSTD-NDVI lag1 (online supplemental figure S2 and table S3). Given these findings, we present results from the ZINB model with rainfall lag1 and LSTD-NDVI lag1 for the village-level analysis in the subsequent sections.

**Table 1:** Assessing the effects of climatic and non-climatic factors on under-five malaria mortality at the health facility catchment level using a negative binomial model: western Kenya Health and Demographic Surveillance System, 2008–2019

Variable	Lags				
	1*, 0†	1*, 1†	2*, 0†	2*, 1†	2*, 2†
	MRR (95% BCI)	MRR (95% BCI)	MRR (95% BCI)	MRR (95% BCI)	MRR (95% BCI)
Fixed effects					
Rainfall	1.14 (1.04, 1.24)‡	1.13 (1.05, 1.22)‡	1.07 (0.99, 1.16)	1.03 (0.94, 1.12)	1.11 (1.02, 1.20)‡
Temperature (LSTD)	0.96 (0.85, 1.09)	1.08 (0.96, 1.21)	0.99 (0.87, 1.12)	1.06 (0.94, 1.19)	1.08 (0.96, 1.22)
NDVI	1.01 (0.88, 1.15)	1.16 (1.03, 1.31)‡	1.09 (0.96, 1.23)	1.18 (1.04, 1.35)‡	1.04 (0.92, 1.18)
Per cent crop cover	1.19 (1.09, 1.30)‡	1.19 (1.09, 1.29)‡	1.19 (1.09, 1.30)‡	1.19 (1.09, 1.30)‡	1.20 (1.10, 1.31)‡
Per cent surface water	1.08 (0.98, 1.17)	1.08 (0.99, 1.17)	1.08 (0.98, 1.17)	1.08 (0.99, 1.17)	1.09 (0.99, 1.18)
Altitude	0.85 (0.78, 0.92)‡	0.83 (0.77, 0.90)‡	0.84 (0.77, 0.91)‡	0.84 (0.77, 0.91)‡	0.86 (0.79, 0.93)‡
Distance to streams (km)	0.93 (0.87, 1.01)	0.93 (0.87, 1.00)‡	0.93 (0.87, 1.00)‡	0.93 (0.87, 1.00)‡	0.94 (0.87, 1.01)
Bed net use	0.84 (0.69, 1.05)	0.82 (0.70, 1.00)‡	0.84 (0.70, 1.01)	0.85 (0.71, 1.03)	0.88 (0.73, 1.07)
iCCM	0.54 (0.35, 1.11)	0.64 (0.39, 1.32)	0.56 (0.36, 1.02)	0.60 (0.38, 1.46)	0.58 (0.35, 1.26)
Socioeconomic status	0.98 (0.93, 1.04)	0.98 (0.93, 1.04)	0.98 (0.93, 1.04)	0.98 (0.93, 1.04)	0.98 (0.92, 1.04)
Time to hospital (hours)	0.99 (0.94, 1.03)	0.98 (0.93, 1.03)	0.98 (0.94, 1.03)	0.98 (0.93, 1.03)	0.98 (0.94, 1.03)
Other parameters					
Spatial variance	0.10 (0.07, 0.14)	0.09 (0.07, 0.13)	0.10 (0.07, 0.13)	0.09 (0.07, 0.13)	0.10 (0.07, 0.13)
Temporal variance	0.31 (0.25, 0.38)	0.29 (0.24, 0.37)	0.32 (0.26, 0.39)	0.31 (0.25, 0.38)	0.32 (0.25, 0.39)
Temporal correlation, (ρ)	0.77 (0.56, 0.97)	0.83 (0.61, 0.97)	0.78 (0.57, 0.94)	0.82 (0.61, 0.97)	0.81 (0.59, 0.97)
Dispersion	26.3 (9.65, 173.26)	25.1 (9.49, 165.14)	25.02 (9.41, 175.65)	24.04 (9.19, 147.57)	25.16 (9.51, 173.58)
DIC	10 353	10 349	10 358	10 353	10 364

* Rainfall.

† LSTD and NDVI. Lags—0, 1 and 2 correspond to current, previous 1 and 2 months, respectively.

‡ Statistically important (significant).

BCI, Bayesian credible interval; DIC, deviance information criterion; iCCM, integrated community case management; LSTD, daytime land surface temperature; MRR, mortality rate ratio; NDVI, Normalized Difference Vegetation Index.

### The effect of climatic and non-climatic variables on malaria mortality

At the health facility level, an increase in rainfall, NDVI and crop cover was associated with an increase in mortality (mortality rate ratio (MRR)=1.13, 95% Bayesian credible interval (BCI): 1.05–1.22), (MRR=1.16, 95% BCI: 1.03–1.31) and (MRR=1.19, 95% BCI: 1.09–1.29), respectively (table 1). Increase in bed net use (MRR=0.82, 95% BCI: 0.70–1.00), distance to the nearest streams (MRR=0.93, 95% BCI: 0.87–1.00) and higher altitude (MRR=0.83, 95% BCI: 0.77–0.90) were associated with lower mortality (table 1). Crop cover had the strongest positive effect.

At the village level, we observed that rainfall (MRR=1.12, 95% BCI: 1.04–1.20, NDVI (MRR=1.16, 95% BCI: 1.06–1.28), crop cover (MRR=1.17, 95% BCI: 1.11–1.24) and time to the hospital (MRR=1.09, 95% BCI: 1.04–1.13) were positively associated with malaria mortality (table 2). On the other hand, bed net use (MRR=0.83, 95% BCI: 0.70–1.00), distance to the nearest streams (MRR=0.89, 95% BCI: 0.83–0.96), altitude (MRR=0.86, 95% BCI: 0.81–0.90) and SES (MRR=0.95, 95% BCI: 0.91–1.00) were found to be protective (table 2).

**Table 2:** Assessing the effects of climatic and non-climatic factors on under-five malaria mortality at the village level using a zero-inflated negative binomial model: western Kenya Health and Demographic Surveillance System, 2008–2019

Variable	Lags				
	1*, 0†	1*, 1†	2*, 0†	2*, 1†	2*, 2†
	MRR (95% BCI)	MRR (95% BCI)	MRR (95% BCI)	MRR (95% BCI)	MRR (95% BCI)
Fixed effects					
Rainfall	1.11 (1.02, 1.20)‡	1.12 (1.04, 1.20)‡	1.05 (0.98, 1.13)	1.02 (0.95, 1.11)	1.09 (1.01, 1.17)‡
Temperature (LSTD)	0.99 (0.88, 1.10)	1.08 (0.98, 1.20)	0.99 (0.89, 1.10)	1.06 (0.95, 1.17)	1.09 (0.98, 1.20)
NDVI	1.06 (0.96, 1.18)	1.16 (1.06, 1.28)‡	1.10 (1.00, 1.22)‡	1.17 (1.06, 1.30)‡	1.07 (0.97, 1.18)
Per cent crop cover	1.18 (1.11, 1.25)‡	1.17 (1.11, 1.24)‡	1.17 (1.11, 1.24)‡	1.17 (1.11, 1.24)‡	1.18 (1.12, 1.25)‡
Per cent surface water	1.01 (0.95, 1.05)	1.01 (0.95, 1.06)	1.01 (0.95, 1.05)	1.01 (0.96, 1.06)	1.01 (0.95, 1.06)
Altitude	0.86 (0.82, 0.91)‡	0.86 (0.81, 0.90)‡	0.86 (0.82, 0.91)‡	0.86 (0.82, 0.91)‡	0.88 (0.83, 0.93)‡
Distance to streams (km)	0.88 (0.82, 0.95)‡	0.89 (0.83, 0.96)‡	0.89 (0.82, 0.96)‡	0.89 (0.83, 0.96)‡	0.89 (0.83, 0.96)
Bed net use	0.84 (0.71, 1.04)	0.84 (0.70, 1.00)‡	0.86 (0.71, 1.03)	0.84 (0.71, 1.03)	0.88 (0.74, 1.05)
iCCM	0.57 (0.35, 1.18)	0.63 (0.39, 1.48)	0.58 (0.37, 1.16)	0.62 (0.39, 1.16)	0.57 (0.36, 1.12)
Socioeconomic status	0.95 (0.91, 0.99)‡	0.95 (0.91, 1.00)‡	0.95 (0.91, 0.99)‡	0.95 (0.91, 0.99)‡	0.95 (0.91, 0.99)‡
Time to hospital (hours)	1.09 (1.04, 1.14)‡	1.09 (1.04, 1.13)‡	1.09 (1.04, 1.13)‡	1.09 (1.04, 1.13)‡	1.09 (1.05, 1.14)‡
Zero-inflated mixing proportion	0.06 (0.00, 0.16)	0.04 (0.00, 0.21)	0.02 (0.00, 0.14)	0.05 (0.00, 0.15)	0.03 (0.00, 0.19)
Other parameters					
Spatial variance	0.11 (0.09, 0.13)	0.11 (0.09, 0.13)	0.11 (0.09, 0.13)	0.11 (0.08, 0.13)	0.11 (0.09, 0.13)
Temporal variance	0.31 (0.25, 0.38)	0.29 (0.24, 0.36)	0.32 (0.26, 0.39)	0.31 (0.25, 0.38)	0.32 (0.26, 0.39)
Temporal correlation, (ρ)	0.77 (0.56, 0.95)	0.82 (0.62, 0.97)	0.78 (0.56, 0.93)	0.80 (0.61, 0.94)	0.79 (0.57, 0.95)
Dispersion	17.26 (4.32, 166.19)	16.4 (4.05, 168.81)	13.87 (3.91, 148.52)	15.75 (4.13, 185.05)	15.32 (3.92, 176.93)
DIC	41 487	37 592	44 589	37 648	57 614

* Rainfall.

† LSTD and NDVI. Lags—0, 1 and 2 correspond to current, previous 1 and 2 months, respectively.

‡ Statistically important (significant).

BCI, Bayesian credible interval; DIC, deviance information criterion; iCCM, integrated community case management; LSTD, daytime land surface temperature; MRR, mortality rate ratio; NDVI, Normalized Difference Vegetation Index.

Rainfall, NDVI, crop cover, bed net use, altitude and distance to streams were important predictors at both levels. However, the village-level analysis found additional variables, including SES and time to the hospital, to be important predictors. LSTD, surface water and iCCM were not statistically important in this analysis. The temporal variation in malaria mortality was higher than the spatial variation in all the models.

### Space-time patterns of malaria mortality

**Figure 4:**
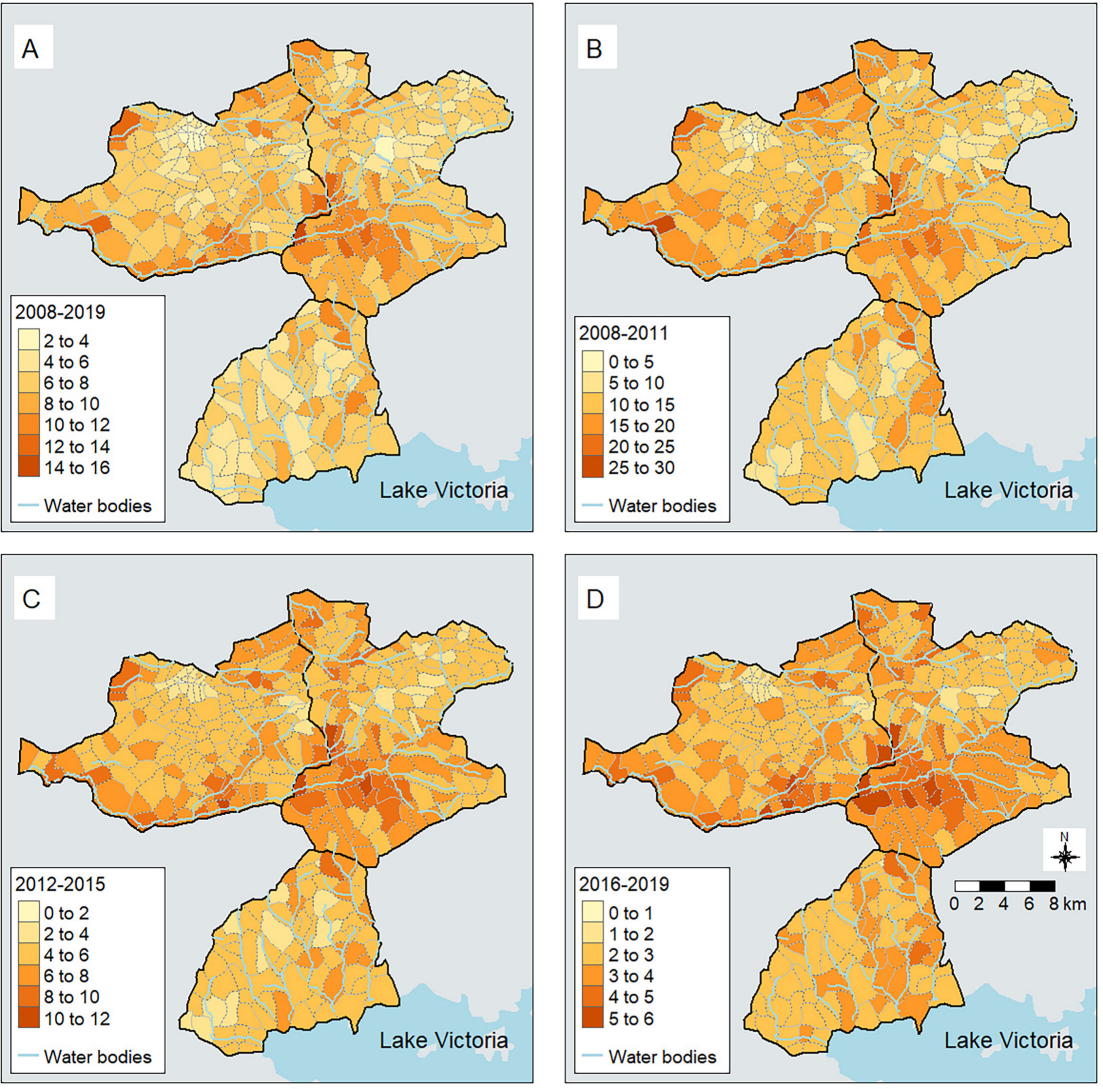
Spatiotemporal patterns of under-five malaria-specific mortality per 1000 person-years in the HDSS during 2008-2019 (A), 2008-2011 (B), 2012–2015 (C) and 2016–2019 (D).

Maps of under-five malaria mortality estimated from the Bayesian spatiotemporal ZINB model for 2008–2019, 2008–2011, 2012–2015 and 2016–2019 are presented in figure 4. There was heterogeneity in malaria mortality within the western Kenya HDSS with villages close to streams, especially along River Yala, having relatively higher malaria mortality compared to other villages. Though mortality significantly reduced over time, the spatial patterns remained relatively similar.

## Discussion

This work highlights the spatial and temporal patterns of malaria mortality in the KEMRI-CDC HDSS during 2008–2019. We observed a steady decline in all-cause and malaria-specific mortality from 2008 to 2011 and a slower decline thereafter in the entire HDSS. This study also observed heterogeneous distribution of malaria mortality in space, though villages with high mortality remained the same during the different time periods. We also found that increase in rainfall, NDVI, crop cover and distance to the nearest health facility were positively associated with malaria mortality while increase in bed net use, SES, distance to streams and altitude were negatively associated with malaria mortality. We observed that there was loss of significance in some effects when data were aggregated at the health facility catchment level.

Space-time models have been used to relate malaria mortality to malaria transmission dynamics within the HDSS.^5 17 18^ However, these studies did not produce mortality maps that can help with targeting control measures in small areas. A study within the HDSS estimated the space-time patterns of entomological inoculation rate and observed space-time (month) patterns in two of the three areas within the HDSS (Gem and Asembo), similar to this study.^43^ Our analysis starts in 2008 when mortality was very high, this is similar to observations by Khagayi *et al* in the same study area where there was a rebound in mortality during 2008.^18^ The high mortality in this year may be associated with the 2007–2008 post election violence that disrupted health systems.^44^ Additionally, we saw high mortality rates in 2009 and gradual reduction in the years that followed.

On average, we observed more deaths in Gem villages that we associate with optimal climatic conditions and the presence of more streams and rivers in Gem compared with the other HDSS areas, which sustain mosquito breeding sites during low transmission seasons. Furthermore, we found that proximity to streams was associated with higher mortality. The slow-moving or stagnant sections of the streams and the flooded/irrigated areas close to the streams provide ideal breeding grounds for anopheles mosquitoes, thus increasing their densities around the streams. Individuals living close to streams are therefore at increased risk of the disease and death because they are more likely to interact with the vector. Since bed net coverage and use is homogeneous within the HDSS, these findings highlight areas where other control measures such as IRS, larval source management, community education on symptoms, risks and prevention of malaria, and targeted mass testing and treatment can be implemented for further reduction in malaria mortality within this setting. Especially when resources are limited, but maximum impact is required. Attractive targeted sugar baits (ATSBs) and spatial repellents may also offer new vector control opportunities which can be employed in these villages.^45^

Compared with aggregated data at health facility catchment level, our study found that fitting models at the village level captured the effects of SES and travel time to the facilities, alongside variables that were statistically important from the catchment level models. This underscores spatial nuances and emphasises the additional benefit of fitting models at finer spatial resolutions when such data are available.

Increase in rainfall in the previous month was associated with an increase in malaria mortality within the HDSS. There were more deaths in the month of May following heavy rains in April, though typically the rains start in March. The increase is mainly due to increased vector abundance, as rainfall creates suitable breeding grounds for mosquitoes. In our earlier work, we observed high malaria incidence during May–July of every year^35 46^ following heavy rains in the wet season. Similar findings have been reported before.^19 25 47^ The high mortality that occurs 1 month before the incidence peak could be associated with late health seeking due to parental involvement in farming activities. A mix of interventions, including community education, larval source management and effective malaria vaccines and monoclonal antibodies, can help reduce these deaths.

Temperature is an important driver of malaria dynamics. Temperature ranges of 20–30°C have been shown to favour mosquito larvae survival, while 25°C is associated with quicker sporogony of *Plasmodium falciparum*, estimated at 12–14 days. Temperatures reduce to this favourable level during the wet season (April–July) in western Kenya, thus creating a conducive environment for mosquitoes to thrive. This reduction in temperature is associated with high mosquito density,^43^ leading to high malaria mortality observed in this study and other previous studies^19 48^ in this setting. Increase in temperature may seem to favour the fight against malaria as increased temperatures are unsuitable for the vector; however, climate projections show that with global warming, both temperature and rainfall will increase in this region.^35^ The effect of temperature was not significant in this analysis. This is possibly due to inclusion of NDVI, a proxy of both rainfall and temperature, in the models and had a stronger effect.

This study observed a steady increase in bed net coverage and use over time. Similar trends were observed in a recent study in a nearby area of Nyakach subcounty, Kisumu county—western Kenya and elsewhere.^49 50^ This is mainly attributed to mass ITN distribution campaigns in the HDSS in 2006, 2011 and 3–4 years thereafter.^50^ We found that increase in bed net use was associated with the reduction of malaria mortality. Other studies reported that bed net use was associated with a reduction in malaria-associated morbidity and mortality in this setting^14 15^ and elsewhere.^51 52^ However, Khagayi *et al* found no association between bed net use and malaria mortality in the same area,^18^ a finding that may be explained by data aggregation. This finding supports sustained systematic distribution and use of bed nets, especially long-lasting insecticidal nets (LLINs). Unfortunately, this is threatened by reduction in funding,^53^ though the Ministry of Health in Kenya plans to mobilise resources within the national and county governments in Kenya.^3^ Mosquito resistance to insecticides also threatens these gains, but new types of insecticide treated nets could reduce the risks.^54 55^

The decline in malaria mortality may also be attributed to the implementation of IMCI^6^ and iCCM^7^ policies adopted by Kenya in 1996/1997 and 2013, respectively, and the establishment of more health facilities in the study area. However, the contribution of these policies to the observed reduction may be minimal as adherence rates to the IMCI policy requirements remain suboptimal,^56 57^ and the iCCM policy was adopted in 2013 after a remarkable reduction in mortality was already observed. Moreover, this study did not observe a significant reduction in malaria mortality after the introduction of iCCM. This may be due to the fact that less than 2% of febrile children aged<5 years seek care from CHWs.^26 58^

We found that the risk of malaria mortality increased with an increase in travel time to the nearest health facility. This increase is directly proportional to distance to the nearest health facility. This finding aligns with similar studies.^18 20 59^ However, Ombok *et al* found no association between distance to the nearest health facility and mortality, and attributed the finding to use of straight-line distances.^16^ Patients are more likely to seek timely care when health facilities are nearer to their homes. Therefore, the additional health facilities established could have played a big role in the reduction of mortality given that in Kenya care is sought from public dispensaries and health centres for majority (45%) of children with febrile illness.^26^ Further reduction in malaria mortality is expected with increase in number of health facilities, which will shorten travel time for health seeking. This will also accelerate the realisation of the second objective of the KMS 2019–2023,^3^ which aims to ensure that 100% of malaria cases are managed through diagnosis and treatment using both facility and community based case management.

Similar to other studies, we found low altitude and low SES to be associated with high malaria mortality.^5 20^ Low-level and middle-level altitudes have previously been associated with higher vector abundance and malaria mortality.^16 60^ Individuals from low SES are more likely to stay in poorly constructed houses, and though there is equity in ITN coverage and use, the houses are likely to have open eaves allowing mosquitoes to rest inside the houses. With persistence in indoor malaria transmission regardless of use of LLINs,^61^ other vector control methods would benefit this subset of the population. Moreover, these children are less likely to access effective treatment for malaria,^62^ therefore addressing socioeconomic inequities could also help towards the goal of malaria elimination in Kenya.

When interpreting our results, it is important to note a few limitations. First, we used VA to determine COD, which may not be the optimal method due to its slightly lower specificity compared with other methods.^17 63^ However, it remains the most viable option for assigning COD at the community level.^31^ Additionally, we did not consider distances and travel time to the nearest private health facilities. Although many residents in this area are less likely to seek care from private health facilities for mild diseases, they are likely to do so for severe illness.^64^ Another limitation is that we did not account for the use of antimalarial drugs and IPTp, which are likely to have contributed to the reduction in mortality.^25^ Since ACT use was introduced in 2006 before the start of this study and IPTp data were unavailable, we assumed that the implementation of these methods was similar in the study area over time, as all the villages are in Siaya county, and health is managed uniformly. We did not include post-2013 spatially varying bed net use data, and this could potentially affect our findings. However, since no significant difference in bed net use was observed in the HDSS villages and the modelling approach employed was rigorous, our findings remain valid. In this analysis, we used reanalysed/downscaled climatic data. This is because obtaining observed climatic data at the desired spatial scale was impossible due to the absence of weather stations in the area.^65^ As weather stations become available, future analyses should consider validating the remotely sensed data against the observed data.

## Conclusion

In the KEMRI-CDC HDSS, under-five year malaria mortality peaks in May. Despite the relatively small size of the area, there is spatial variation in mortality. This information could be helpful to the malaria control programme and the Siaya County health department when deciding when and where to deploy control interventions in this area. Bed net use remains a viable tool in the fight against malaria and towards malaria elimination, therefore efforts towards acquisition of adequate funding is necessary to sustain the mass ITN distribution campaigns. Furthermore, these findings highlight the time delays between climate suitability with mortality and the importance of fine spatial scale analysis when data are available.

## Supplementary material

10.1136/bmjgh-2023-014614 online supplemental file 1

10.1136/bmjgh-2023-014614 online supplemental file 2

## Data Availability

Data are available on reasonable request.

## References

[1] World health organization (2022). World malaria report 2022.

[2] Macharia PM, Giorgi E, Noor AM (2018). Spatio-temporal analysis of Plasmodium falciparum prevalence to understand the past and chart the future of malaria control in Kenya. Malar J.

[3] Ministry of Health - Kenya (2019). Kenya malaria strategy 2019-2023.

[4] Desai M, Buff AM, Khagayi S (2014). Age-specific malaria mortality rates in the KEMRI/CDC health and demographic surveillance system in western Kenya, 2003-2010. PLoS ONE.

[5] Khagayi S, Amek N, Bigogo G (2017). Bayesian spatio-temporal modeling of mortality in relation to malaria incidence in Western Kenya. PLoS ONE.

[6] Rakha MA, Abdelmoneim A-NM, Farhoud S (2013). Does implementation of the IMCI strategy have an impact on child mortality? A retrospective analysis of routine data from Egypt. BMJ Open.

[7] Juma PA, Owuor K, Bennett S (2015). Integrated community case management for childhood illnesses: explaining policy resistance in Kenya. Health Policy Plan.

[8] Macharia PM, Joseph NK, Sartorius B (2021). Subnational estimates of factors associated with under-five mortality in Kenya: a spatio-temporal analysis, 1993-2014. BMJ Glob Health.

[9] Thomson MC, Ukawuba I, Hershey CL (2017). Using Rainfall and Temperature Data in the Evaluation of National Malaria Control Programs in Africa. Am J Trop Med Hyg.

[10] Zbinden ZD (2020). Temporal dynamics of stream fish assemblages and the role of spatial scale in quantifying change. Ecol Evol.

[11] Clark LP, Harris MH, Apte JS (2022). National and Intraurban Air Pollution Exposure Disparity Estimates in the United States: impact of Data-Aggregation Spatial Scale. Environ Sci Technol Lett.

[12] Raghavan RK, Brenner KM, Harrington JA (2013). Spatial scale effects in environmental risk-factor modelling for diseases. Geospat Health.

[13] Amek NO, Odhiambo FO, Khagayi S (2014). Childhood cause-specific mortality in rural Western Kenya: application of the InterVA-4 model. Glob Health Action.

[14] Phillips-Howard PA, Nahlen BL, Kolczak MS (2003). Efficacy of permethrin-treated bed nets in the prevention of mortality in young children in an area of high perennial malaria transmission in western Kenya. Am J Trop Med Hyg.

[15] ter Kuile FO, Terlouw DJ, Phillips-Howard PA (2003). Impact of permethrin-treated bed nets on malaria and all-cause morbidity in young children in an area of intense perennial malaria transmission in western Kenya: cross-sectional survey. Am J Trop Med Hyg.

[16] Ombok M, Adazu K, Odhiambo F (2010). Geospatial distribution and determinants of child mortality in rural western Kenya 2002-2005: geospatial distribution and determinants of child mortality in rural western Kenya. Trop Med Int Health.

[17] Amek NO, Van Eijk A, Lindblade KA (2018). Infant and child mortality in relation to malaria transmission in KEMRI/CDC HDSS, Western Kenya: validation of verbal autopsy. Malar J.

[18] Khagayi S, Desai M, Amek N (2019). Modelling the relationship between malaria prevalence as a measure of transmission and mortality across age groups. Malar J.

[19] Sewe M, Rocklöv J, Williamson J (2015). The association of weather variability and under five malaria mortality in KEMRI/CDC HDSS in Western Kenya 2003 to 2008: a time series analysis. Int J Environ Res Public Health.

[20] Hollowell T, Sewe MO, Rocklöv J (2023). Public health determinants of child malaria mortality: a surveillance study within Siaya County, Western Kenya. Malar J.

[21] Ferrão JL, Mendes JM, Painho M (2017). Malaria mortality characterization and the relationship between malaria mortality and climate in Chimoio, Mozambique. Malar J.

[22] Dasgupta S (2018). Burden of climate change on malaria mortality. Int J Hyg Environ Health.

[23] Panzi EK, Kandala NI, Kafinga EL (2022). Forecasting Malaria Morbidity to 2036 Based on Geo-Climatic Factors in the Democratic Republic of Congo. Int J Environ Res Public Health.

[24] Ssempiira J, Kasirye I, Kissa J (2018). Measuring health facility readiness and its effects on severe malaria outcomes in Uganda. Sci Rep.

[25] Ssempiira J, Kissa J, Nambuusi B (2018). Interactions between climatic changes and intervention effects on malaria spatio-temporal dynamics in Uganda. Parasite Epidemiol Control.

[26] Division of National Malaria Programme (2020). Kenya malaria indicator survey 2020.

[27] National AIDS and STI Control Programme (2022). Kenya population-based hiv impact assessment (kenphia) 2018:final report.

[28] Adazu K, Lindblade KA, Rosen DH (2005). Health and demographic surveillance in rural western Kenya: a platform for evaluating interventions to reduce morbidity and mortality from infectious diseases. Am J Trop Med Hyg.

[29] Odhiambo FO, Laserson KF, Sewe M (2012). Profile: the KEMRI/CDC Health and Demographic Surveillance System--Western Kenya. Int J Epidemiol.

[30] Feikin DR, Olack B, Bigogo GM (2011). The burden of common infectious disease syndromes at the clinic and household level from population-based surveillance in rural and urban Kenya. PLoS ONE.

[31] Byass P, Chandramohan D, Clark SJ (2012). Strengthening standardised interpretation of verbal autopsy data: the new InterVA-4 tool. Glob Health Action.

[32] Wan Z, Hook S, Hulley G (2015). MOD11A2 modis/terra land surface temperature/emissivity 8-day l3 global 1km sin grid v006.

[33] Didan K (2021). MODIS/terra vegetation indices monthly l3 global 1km sin grid v061.

[34] Funk C, Peterson P, Landsfeld M (2015). The climate hazards infrared precipitation with stations--a new environmental record for monitoring extremes. Sci Data.

[35] Nyawanda BO, Beloconi A, Khagayi S (2023). The relative effect of climate variability on malaria incidence after scale-up of interventions in western Kenya: a time-series analysis of monthly incidence data from 2008 to 2019. Parasite Epidemiol Control.

[36] NASA Shuttle Radar Topography Mission (2013). Shuttle radar topography mission (srtm) global.

[37] Buchhorn M, Lesiv M, Tsendbazar N-E (2020). Copernicus Global Land Cover Layers—Collection 2. Remote Sens (Basel).

[38] Pekel J-F, Cottam A, Gorelick N (2016). High-resolution mapping of global surface water and its long-term changes. Nat New Biol.

[39] Melo RA, Zarruk D (2023). Distance and travel time between two points from google maps version 4.0.4. https://github.com/jlacko/gmapsdistance.

[40] Amek N, Vounatsou P, Obonyo B (2015). Using health and demographic surveillance system (HDSS) data to analyze geographical distribution of socio-economic status; an experience from KEMRI/CDC HDSS. Acta Trop.

[41] Amin AA, Zurovac D, Kangwana BB (2007). The challenges of changing national malaria drug policy to artemisinin-based combinations in Kenya. Malar J.

[42] R Foundation for Statistical Computing R: a language and environment for statistical computing.

[43] Amek N, Bayoh N, Hamel M (2012). Spatial and temporal dynamics of malaria transmission in rural Western Kenya. Parasit Vectors.

[44] Feikin DR, Adazu K, Obor D (2010). Mortality and health among internally displaced persons in western Kenya following post-election violence, 2008: novel use of demographic surveillance. Bull World Health Organ.

[45] Omondi S, Kosgei J, Agumba S (2022). Natural sugar feeding rates of Anopheles mosquitoes collected by different methods in western Kenya. Sci Rep.

[46] Beloconi A, Nyawanda BO, Bigogo G (2023). Malaria, climate variability, and interventions: modelling transmission dynamics. Sci Rep.

[47] Kipruto EK, Ochieng AO, Anyona DN (2017). Effect of climatic variability on malaria trends in Baringo County, Kenya. Malar J.

[48] Sewe MO, Ahlm C, Rocklöv J (2016). Remotely Sensed Environmental Conditions and Malaria Mortality in Three Malaria Endemic Regions in Western Kenya. PLoS ONE.

[49] Kamau A, Musau M, Mtanje G (2022). Long-lasting insecticide-treated net use and malaria infections on the Kenyan coast. Trans R Soc Trop Med Hyg.

[50] Ng’ang’a PN, Aduogo P, Mutero CM (2021). Long lasting insecticidal mosquito nets (LLINs) ownership, use and coverage following mass distribution campaign in Lake Victoria basin, Western Kenya. BMC Public Health.

[51] Demombynes G, Trommlerová SK (2016). What has driven the decline of infant mortality in Kenya in the 2000s?. Econ Hum Biol.

[52] Eckert E, Florey LS, Tongren JE (2017). Impact Evaluation of Malaria Control Interventions on Morbidity and All-Cause Child Mortality in Rwanda, 2000-2010. Am J Trop Med Hyg.

[53] Zelman B, Kiszewski A, Cotter C (2014). Costs of eliminating malaria and the impact of the global fund in 34 countries. PLoS ONE.

[54] Owuor KO, Machani MG, Mukabana WR (2021). Insecticide resistance status of indoor and outdoor resting malaria vectors in a highland and lowland site in Western Kenya. PLoS ONE.

[55] Meiwald A, Clark E, Kristan M (2022). Association of Reduced Long-Lasting Insecticidal Net Efficacy and Pyrethroid Insecticide Resistance With Overexpression of CYP6P4, CYP6P3, and CYP6Z1 in Populations of Anopheles coluzzii From Southeast Côte d’Ivoire. J Infect Dis.

[56] Krüger C, Heinzel-Gutenbrunner M, Ali M (2017). Adherence to the integrated management of childhood illness guidelines in Namibia, Kenya, Tanzania and Uganda: evidence from the national service provision assessment surveys. BMC Health Serv Res.

[57] Deichsel EL, Keita AM, Verani JR (2023). Management of Diarrhea in Young Children in Sub-Saharan Africa: Adherence to World Health Organization Recommendations During the Global Enteric Multisite Study (2007-2011) and the Vaccine Impact of Diarrhea in Africa (VIDA) Study (2015-2018). Clin Infect Dis.

[58] National Malaria Control Programme, Kenya National Bureau of Statistics (KNBS), ICF International (2016). Kenya malaria indicator survey 2015.

[59] Karra M, Fink G, Canning D (2016). Facility distance and child mortality: a multi-country study of health facility access, service utilization, and child health outcomes. Int J Epidemiol.

[60] Kasasa S, Asoala V, Gosoniu L (2013). Spatio-temporal malaria transmission patterns in Navrongo demographic surveillance site, northern Ghana. Malar J.

[61] Machani MG, Ochomo E, Amimo F (2020). Resting behaviour of malaria vectors in highland and lowland sites of western Kenya: implication on malaria vector control measures. PLoS ONE.

[62] Were V, Buff AM, Desai M (2019). Trends in malaria prevalence and health related socioeconomic inequality in rural western Kenya: results from repeated household malaria cross-sectional surveys from 2006 to 2013. BMJ Open.

[63] Rakislova N, Jordao D, Ismail MR (2021). Accuracy of verbal autopsy, clinical data and minimally invasive autopsy in the evaluation of malaria-specific mortality: an observational study. BMJ Glob Health.

[64] Emukule GO, Osoro E, Nyawanda BO (2023). Healthcare-seeking behavior for respiratory illnesses in Kenya: implications for burden of disease estimation. BMC Public Health.

[65] Coughlan de Perez E, Arrighi J, Marunye J (2023). Challenging the universality of heatwave definitions: gridded temperature discrepancies across climate regions. Clim Change.

