# Supplementary material for: The effects of climatic and non-climatic factors on malaria mortality at different spatial scales in western Kenya, 2008–2019
Source: BMJ Glob Health. 2024 Sep 7;9(9):e014614. doi: 10.1136/bmjgh-2023-014614 (PMC11381700; doi:10.1136/bmjgh-2023-014614)
Supplement: online supplemental file 1 [file bmjgh-9-9-s001.pdf]

Supplemental File 1: Bayesian model formulation

Let y_{it} be the number of observed malaria deaths in village i=1,2…384 at time t=1,2…144 (12 months for 12 years). We assumed that y_{it} followed a zero inflated negative binomial distribution (ZINB) with a mixture of two components; one corresponding to the negative binomial distribution, the other modelling the excess zeros that were observed with frequencies higher than those expected by the NB, that is:

$$f(Y_{it} = y_{it}) = \begin{cases} p_{it} + (1 - p_{it})\left(\dfrac{r}{r + \mu_{it}}\right)^r & , \quad y_{it} = 0 \\ (1 - p_{it})\dfrac{(y_{it} + r - 1)!}{y_{it}!\,(r - 1)!}\left(\dfrac{r}{r + \mu_{it}}\right)^r \left(\dfrac{\mu_{it}}{r + \mu_{it}}\right)^{y_{it}} & , \quad y_{it} \geq 0 \end{cases}$$

where the average number of deaths per village in any given month is given by $(1 - p_{it})\mu_{it}$, r is the dispersion parameter of the NB distribution and p_{it} is the mixing proportion. The log link function was used to relate the mean μ_{it} of the NB distribution with the predictors, i.e.:

$$log(\mu_{it}) = log\,(N_{it}) + X_{it}^T\beta \; + \epsilon_t + \; \omega_i$$

where N_{it} is an offset term defined as the total person years of observation (pyo) in a given village i at time t; β is a vector of regression coefficients for the matrix of predictors X_{it}^T (including climatic factors, interventions and other covariates). We follow a Bayesian formulation and assume that the monthly random effects, ϵ_t, are modelled by a first order autoregressive process AR(1) with temporal variance σ_t^2. ω_i, i=1,2,…,384, are modelled via a conditional autoregressive (CAR) process where each ω_i conditional on the neighbour ω_j follows a normal distribution with mean equal to the average of neighbouring villages ω_j and variance inversely proportional to the number of neighbouring villages n_i; i.e., $\omega_i|\omega_j \sim N(\gamma\sum_{l\in\delta_j}\omega_j, \frac{\sigma_\omega^2}{n_i})$, where γ quantifies the amount of spatial correlation present in the data, and σ_ω^2 measures the spatial variance. ω_i and ω_j are adjacent villages in the set of all adjacent villages δ_i. Similar formulation was used for the health facility catchment level model with ω_i, i=1,2,…,48.

The Bayesian model formulation requires specification of prior distributions. Therefore, for the regression coefficients non-informative priors from the normal distribution with mean 0 and variance of 100, that is $\beta \sim N(0,100)$, were specified whereas priors from Gamma distribution with mean 0 and variance 100 was used for the dispersion parameter, r. For the temporal random effects ϵ_t, which are considered to be temporally correlated, we assumed that $\epsilon_1 \sim N(0, \frac{\sigma^2}{1-\rho^2})$ and $\epsilon_t \sim N(\rho\epsilon_{t-1}, \sigma^2$ for t=2,3…144 and that ρ is the autocorrelation parameter with a uniform prior distribution i.e., $\rho \sim U(-1,1)$. The priors for the temporal and spatial random effects variance, σ_t^2 and σ_ω^2, were from the inverse Gamma distribution with mean 10 and variance 100 i.e., $\sigma_t^2, \sigma_\omega^2 \sim IG(0.1,0.01)$.. Through Markov chain Monte Carlo (MCMC) simulation, we estimated the model parameters by fitting (zero inflated) negative binomial Bayesian models in Just Another Gibbs Sampler (JAGS) software [1]. We ran two chains of 50,000 iterations, each with a burn-in of 5,000 and assessed convergence using density plots, trace plots, and the Gelman-Rubin diagnostics [2] using coda in R software.

References

1 Plummer M. JAGS: A Program for Analysis of Bayesian Graphical Models Using Gibbs Sampling. *Proc 3rd Int Workshop Distrib Stat Comput*. 2003.

2 Brooks SP, Roberts GO. Convergence assessment techniques for Markov chain Monte Carlo. *Stat Comput*. 1998;8:319–35.
