# Supplementary material for: The effects of climatic and non-climatic factors on malaria mortality at different spatial scales in western Kenya, 2008–2019
Source: BMJ Glob Health. 2024 Sep 7;9(9):e014614. doi: 10.1136/bmjgh-2023-014614 (PMC11381700; doi:10.1136/bmjgh-2023-014614)
Supplement: online supplemental file 2 [file bmjgh-9-9-s002.pdf]

Supplemental File 2: Figures and Tables

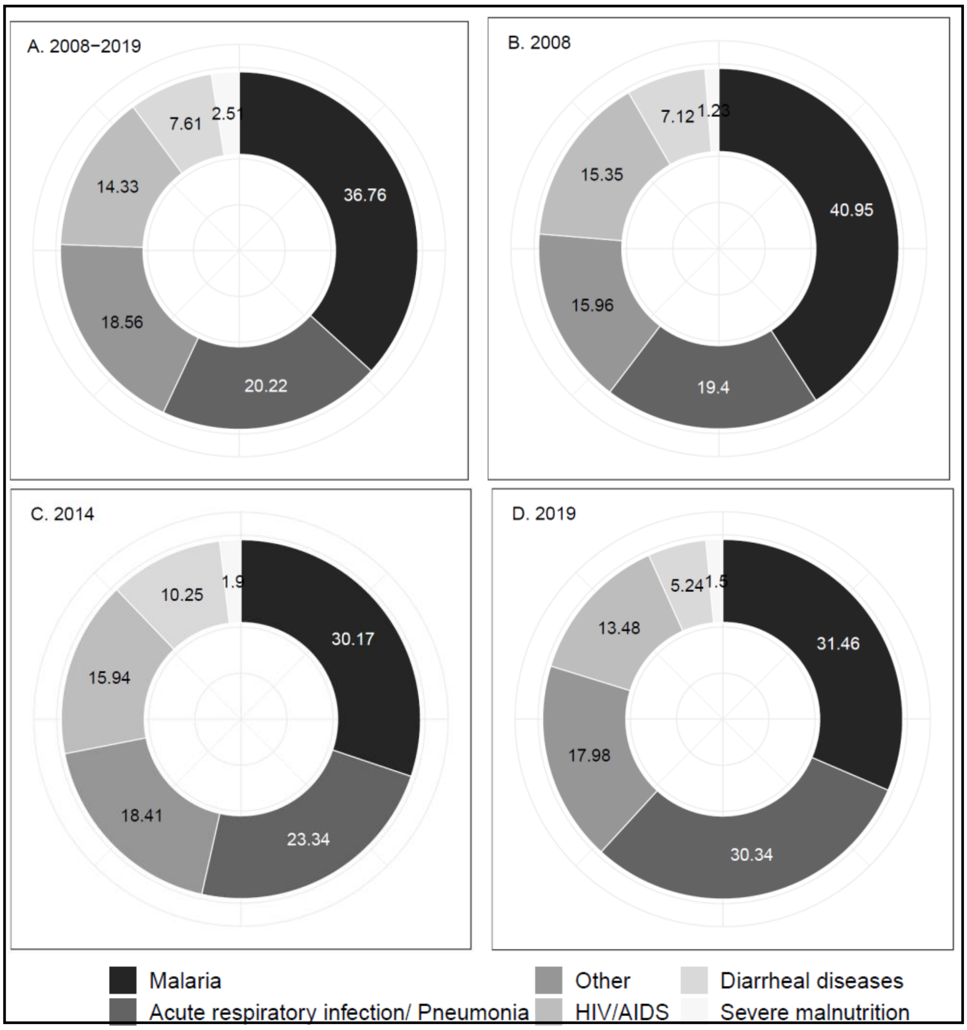

Figure S1: Relative frequencies of the leading causes of death among children under 5 years old in the KEMRI-CDC HDSS during the period 2008-2019 and at specific years.

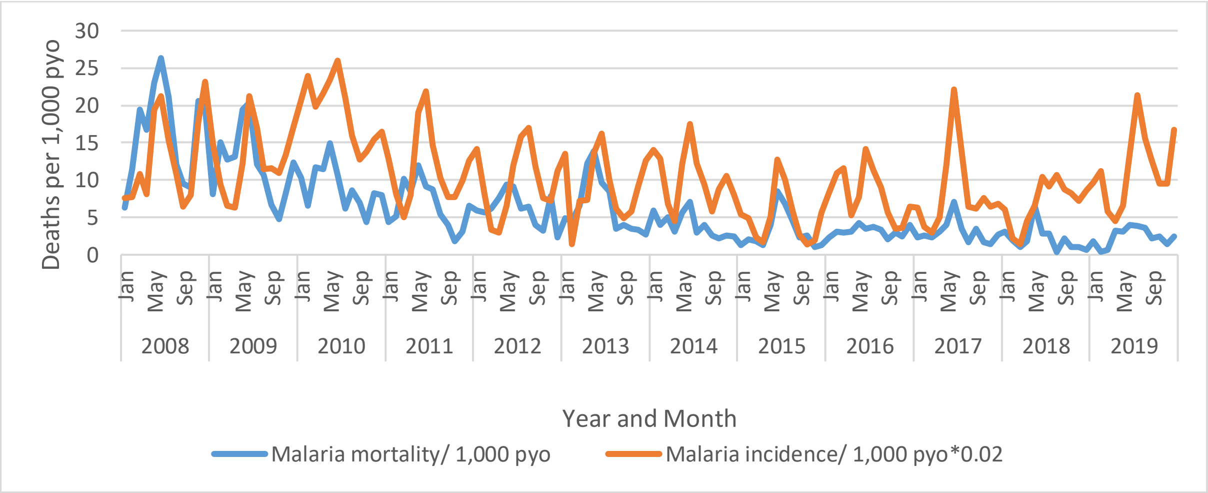

Figure S2: Time series plot comparing malaria mortality and incidence peaks in the KEMRI-CDC HDSS, 2008-2019

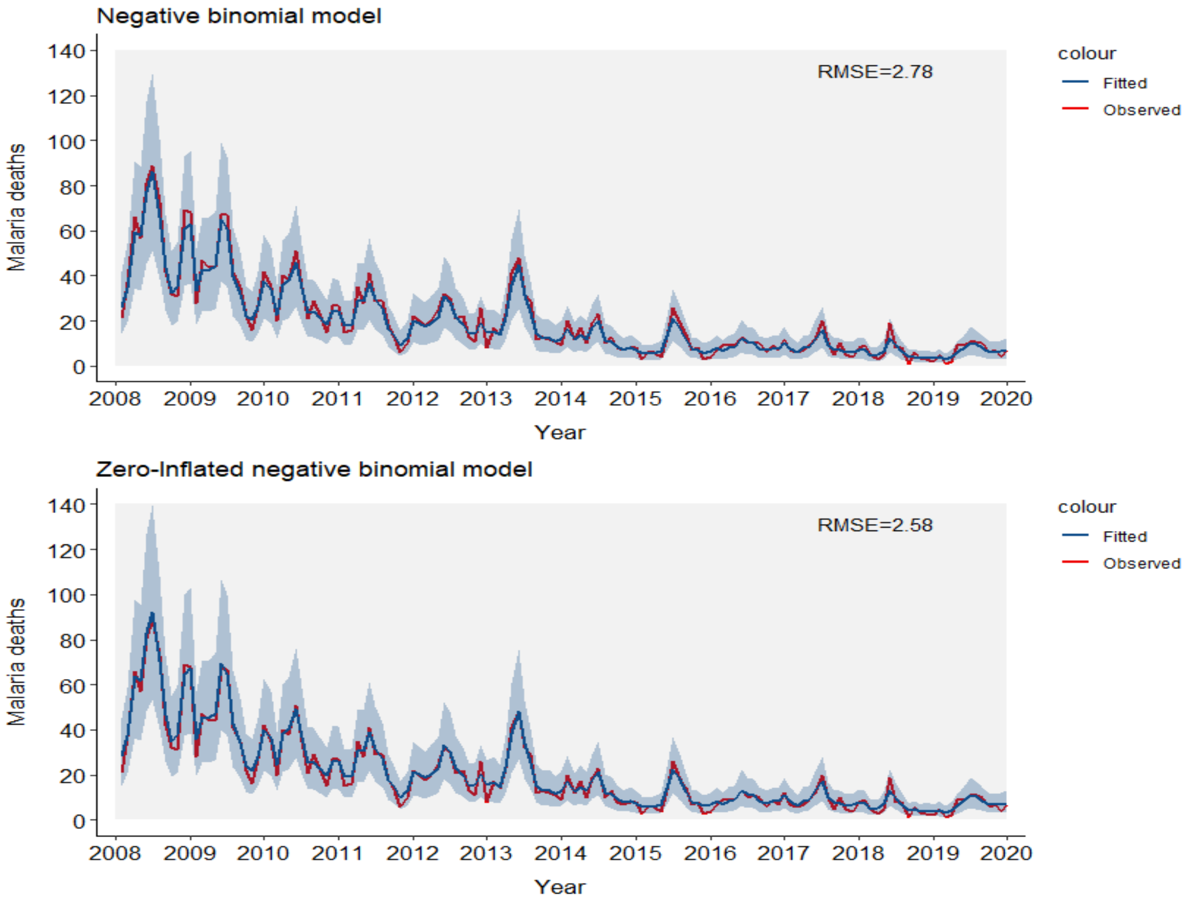

Figure S3: Observed (red line), posterior median (blue line), and 95% Bayesian credible intervals around the posterior median (blue shaded area) using the negative binomial and zero-inflated negative binomial models on village level data, 2008-2019.

Table S1: Estimates of the effects of rainfall and Land Surface Day Temperature (LSTD) on under-five malaria mortality for different time lags based on Bayesian spatio-temporal models.

Variable		LSTD			NDVI		
	Mortality	Lag 0	Lag 1	Lag 2	Lag 0	Lag 1	Lag 2
**Health facility catchment level**							
**Mortality**		-0.08	-0.04	0.03	0.05	0.05	-0.01
**Rain**							
**Lag 0**	0.00	-0.44	0.13	0.33	0.48	-0.10	-0.39
**Lag 1**	0.05	-0.62	-0.44	0.14	0.69	0.48	-0.11
**Lag 2**	0.03	-0.50	-0.62	-0.44	0.43	0.69	0.47
**NDVI**							
**Lag 0**	0.05	-0.84	-0.51	-0.04			
**Lag 1**	0.05	-0.56	-0.84	-0.51			
**Lag 2**	-0.01	-0.13	-0.55	-0.84			
**Village level**							
**Mortality**		-0.03	-0.02	0.01	0.02	0.02	0.00
**Rain**							
**Lag 0**	0.00	-0.44	0.14	0.34	0.47	-0.11	-0.40
**Lag 1**	0.02	-0.62	-0.44	0.14	0.68	0.46	-0.13
**Lag 2**	0.01	-0.49	-0.61	-0.43	0.42	0.67	0.45
**NDVI**							
**Lag 0**	0.02	-0.83	-0.51	-0.04			
**Lag 1**	0.02	-0.54	-0.83	-0.50			
**Lag 2**	0.00	-0.12	-0.54	-0.83			

Rain and lstD_0,1 ,2 correspond to no lag (current month) and lags of 1 and 2 months, respectively;
LSTD- Day time Land Surface Temperature; NDVI- Normalized difference vegetation index

Table S2: The effects of climatic and non-climatic factors on under-five malaria mortality at health facility catchment level using a negative binomial (NB) model: Western Kenya HDSS, 2008-2019.

**Variable**	**Lags**				
	**1[a],0[b]**	**1[a],1[b]**	**2[a],0[b]**	**2[a],1[b]**	**2[a],2[b]**
	MRR [c] (95% BCI)	MRR (95% BCI)	MRR (95% BCI)	MRR (95% BCI)	MRR (95% BCI)
Fixed effects					
Rainfall	**1.14 (1.04,1.24)***	**1.13 (1.04,1.22)***	1.06 (0.98,1.15)	1.03 (0.94,1.13)	**1.11 (1.02,1.21)***
Temperature <25 °C	1	1	1	1	1
25-30 °C	0.76 (0.57,1.03)	0.93 (0.65,1.32)	0.79 (0.58,1.10)	0.91 (0.62,1.27)	0.97 (0.65,1.55)
> 30 °C	0.77 (0.56,1.09)	1.00 (0.69,1.45)	0.81 (0.57,1.16)	0.96 (0.63,1.38)	1.09 (0.71,1.76)
NDVI	1.04 (0.95,1.14)	**1.12 (1.03,1.21)***	**1.10 (1.01,1.19)***	**1.14 (1.04,1.25)***	1.01 (0.93,1.10)
% crop cover	**1.19 (1.09,1.30)***	**1.19 (1.09,1.29)***	**1.19 (1.09,1.30)***	**1.19 (1.09,1.30)***	**1.20 (1.09,1.31)***
% Surface water	1.08 (0.98,1.17)	1.08 (0.98,1.17)	1.08 (0.98,1.17)	1.08 (0.98,1.17)	1.08 (0.99,1.18)
altitude	**0.84 (0.77,0.92)***	**0.83 (0.77,0.90)***	**0.84 (0.77,0.91)***	**0.84 (0.77,0.91)***	**0.86 (0.79,0.94)***
Distance to streams (km)	**0.93 (0.87,1.00)***	**0.93 (0.87,1.00)***	**0.93 (0.87,1.00)***	**0.93 (0.87,1.00)***	0.94 (0.87,1.01)
Bed net use	0.84 (0.70,1.02)	**0.84 (0.71,1.00)***	0.84 (0.71,1.02)	0.84 (0.69,1.02)	0.86 (0.73,1.05)
iCCM	**0.53 (0.35,1.00)***	0.61 (0.38,1.41)	0.58 (0.37,1.37)	0.61 (0.37,1.53)	0.57 (0.35,1.44)
Socio-economic status	0.98 (0.93,1.04)	0.98 (0.93,1.04)	0.98 (0.93,1.04)	0.98 (0.93,1.04)	0.98 (0.92,1.04)
Time to hospital (hrs)	0.98 (0.93,1.03)	0.98 (0.94,1.03)	0.98 (0.93,1.03)	0.98 (0.93,1.03)	0.98 (0.94,1.03)
Other parameters					
Spatial variance	0.10 (0.07,0.13)	0.10 (0.07,0.13)	0.10 (0.07,0.13)	0.09 (0.07,0.13)	0.01 (0.07,0.13)
Temporal variance	0.31 (0.25,0.38)	0.30 (0.24,0.37)	0.32 (0.26,0.39)	0.31 (0.25,0.38)	0.32 (0.26,0.39)
Temporal correlation, (ρ)	0.77 (0.56,0.95)	0.82 (0.61,0.98)	0.78 (0.57,0.96)	0.81 (0.60,0.97)	0.79 (0.58,0.96)
Dispersion	26.48 (9.80,179.22)	24.44 (9.31,146.38)	25.13 (9.47,166.28)	23.72 (9.17,146.71)	25.2 (9.55,168.85)
DIC	10350	10351	10353	10353	10357

[a] Rainfall and [b] LSTD and NDVI lags –0, 1 and 2 correspond to current, previous one and two months respectively; [c] Mortality rate ratio; [d] Integrated community case management; * Statistically important

Table S3: The effects of climatic and non-climatic factors on under-five malaria mortality at the village level using a negative binomial (NB) model: Western Kenya HDSS, 2008-2019

**Variable**	**Lags**				
	**$1^a,0^b$**	**$1^a,1^b$**	**$2^a,0^b$**	**$2^a,1^b$**	**$2^a,2^b$**
	MRR (95% BCI)	MRR (95% BCI)	MRR (95% BCI)	MRR (95% BCI)	MRR (95% BCI)
Fixed effects					
Rainfall	**1.11 (1.02,1.20)***	**1.12 (1.04,1.20)***	1.06 (0.98,1.13)	1.02 (0.94,1.11)	**1.09 (1.01,1.17)***
Temperature (LSTD)	0.98 (0.88,1.10)	1.08 (0.97,1.20)	0.99 (0.89,1.10)	1.06 (0.95,1.17)	1.08 (0.97,1.2)
NDVI	1.06 (0.96,1.18)	**1.16 (1.05,1.28)***	**1.10 (1.00,1.22)***	**1.17 (1.06,1.30)***	1.06 (0.96,1.17)
% crop cover	**1.18 (1.11,1.25)***	**1.17 (1.11,1.24)***	**1.18 (1.11,1.24)***	**1.17 (1.11,1.24)***	**1.18 (1.12,1.25)***
% Surface water	1.01 (0.95,1.05)	1.01 (0.95,1.06)	1.01 (0.95,1.05)	1.01 (0.96,1.06)	1.01 (0.95,1.06)
altitude	**0.86 (0.82,0.91)***	**0.86 (0.81,0.90)***	**0.86 (0.82,0.91)***	**0.87 (0.82,0.91)***	**0.88 (0.83,0.93)***
Distance to streams (km)	**0.88 (0.82,0.95)***	**0.89 (0.83,0.96)***	**0.89 (0.82,0.95)***	**0.89 (0.83,0.96)***	**0.89 (0.82,0.96)***
Bed net use	0.85 (0.71,1.05)	**0.83 (0.69,0.98)***	0.84 (0.71,1.04)	0.85 (0.72,1.04)	0.86 (0.73,1.07)
iCCM	**0.56 (0.36,0.99)***	0.62 (0.39,1.23)	0.57 (0.36,1.05)	0.62 (0.37,1.25)	0.56 (0.35,1.02)
Socio-economic status	**0.95 (0.91,0.99)***	**0.95 (0.91,1.00)***	**0.95 (0.91,0.99)***	**0.95 (0.91,1.00)***	**0.95 (0.91,0.99)***
Time to hospital (hrs)	**1.09 (1.04,1.14)***	**1.09 (1.04,1.13)***	**1.09 (1.04,1.13)***	**1.09 (1.04,1.13)***	**1.09 (1.05,1.14)***
Other parameters					
Spatial variance	0.11 (0.09,0.13)	0.11 (0.09,0.13)	0.11 (0.09,0.13)	0.11 (0.09,0.13)	0.11 (0.09,0.13)
Temporal variance	0.31 (0.25,0.38)	0.30 (0.24,0.36)	0.32 (0.26,0.39)	0.31 (0.25,0.38)	0.32 (0.26,0.39)
Temporal correlation, (ρ)	0.77 (0.57,0.95)	0.81 (0.61,0.95)	0.77 (0.56,0.94)	0.81 (0.61,0.95)	0.76 (0.55,0.94)
Dispersion	9.34 (3.45,114.94)	9.2 (3.39,115.42)	9.39 (3.42,126.87)	9.15 (3.42,95.36)	9.33 (3.44,104.74)
DIC	21100	21097	21115	21107	21120

[a] Rainfall and [b] LSTD and NDVI lags –0, 1 and 2 correspond to current, previous one and two months respectively; [c] Mortality rate ratio; [d] Integrated community case management; * Statistically important
